# The VicHealth Indicators population survey: methodology, prevalence of behavioural risk factors, and use in local policy

**DOI:** 10.1186/s12889-020-09605-5

**Published:** 2020-10-02

**Authors:** Annemarie Wright, Jane Shill, Nikki Honey, Anthony F. Jorm, Bruce Bolam

**Affiliations:** 1 Victorian Health Promotion Foundation, Melbourne, Australia; 2 Melbourne School of Population and Global Health, The University of Melbourne, Melbourne, Australia; 3 Social Research Centre, Melbourne, Australia; 4 Department of Health and Human Services, Victorian State Government, Melbourne, Australia

**Keywords:** Population survey, Illness prevention, Health promotion, Behavioural risk factor, Policy, Practice, NCDs

## Abstract

**Background:**

The Victorian Health Promotion Foundation (VicHealth) is an Australian state-based government agency with a remit to promote health by targeting physical activity, diet, mental wellbeing, tobacco use and alcohol consumption. Population health data is crucial to this work. This paper reports on the measures and methods used in surveillance, examines the prevalence of risk factors in sub-populations and use of risk factor data in local policy and planning.

**Methods:**

The VicHealth Indicators (VHI) cross-sectional population telephone survey of behavioural and attitudinal health risk factors involved interviews with 22,819 respondents aged 18 years+ from the state of Victoria in 2015. Means or percent prevalences (with 95% CIs) of indicators are presented. Statistically significant differences between the state level and sub-population estimates were deemed to exist when confidence intervals of estimates did not overlap. Use of the data in local policy was assessed through an audit of 77 Municipal Public Health and Wellbeing Plans for 2017–2021. Use in municipal plans according to the municipality’s geographical region type and SES was analysed using Welch’s ANOVA.

**Results:**

The average vegetable intake was 2.2 serves per day, far below the national guidelines of 5 serves per day, and only 4 in 10 Victorians were sufficiently active. Young males were twice as likely to be at high risk of alcohol harm compared to the state estimate. Women were twice as likely to feel unsafe walking after dark compared to males. There was a consistent pattern of significantly less favourable results for people living in outer metropolitan areas and a socio-economic gradient was evident for most risk factors. Almost 50% of municipalities used VHI data in their local policy plans. Use of VHI data was significantly higher in high SES municipalities and significantly lower in low SES municipalities relative to the mean.

**Conclusions:**

The findings indicate the need for continued targeted action on behavioural risk factors, particularly diet and physical activity, and that more intensive policy and practice action is required to address health inequities to ensure that all Victorians can experience good health. Increased support for low SES municipality policy planning may be warranted.

## Background

Behavioural risk factors for health, including smoking, alcohol consumption, poor diet and inadequate physical activity, have major health impacts. A recent report on burden of disease in Australia found that behavioural risk factors are the leading contributor to disease burden, and that the burden attributed to these risk factors increases as socio-economic status declines [1]. Internationally, the Global Burden of Diseases, Injuries, and Risk Factors Study 2017 estimates that behavioural risk factors contribute to 21.6% of disease burden globally [2]. Population surveillance of behavioural risk factors is therefore recognised as crucial for informing, monitoring and evaluating population health policies and programs [3, 4].

Indeed, behavioural risk factor surveillance is now common practice in many countries. For example, the US Behavioral Risk Factor Surveillance System (BRFSS), conducted by the Centers for Disease Control and Prevention, is an ongoing, cross-sectional, random-digit–dialed telephone survey to both landline and mobile (cell) phones that completes approximately 400,000 interviews with adults residing in the United States or its territories each year [3]. The validity and reliability of the BRFSS survey has been established due to the use of the survey indicators as comparator benchmarks for a wide array of other studies. It is central to the development of public health policies and programs at a state and local level.

Use of risk factor surveillance in developing countries has been supported by the World Health Organisation (WHO) through their STEPwise approach to Surveillance (STEPS) initiative. STEPS is designed to provide population-level data on behavioural risk factors to inform the development of policies and programs that address increasing rates of non-communicable diseases [4].

In Australia, the Australian Bureau of Statistics (ABS) conducts the National Health Survey, however its sample size of approximately 2500 adults for Victoria [5] is too small to inform programs and policies at a local level. Therefore, many states in Australia have their own approach to behavioural risk factor surveillance. This is one of the reasons that state-level surveys have evolved in the USA alongside the BRFSS, that is, to inform planning for the local context and ensure that health data is rapidly available at the smallest geographical level to address health disparities based on factors such as ethnicity and socioeconomic status [6].

The Victorian Health Promotion Foundation (VicHealth) is a state-based government agency in Victoria, Australia, with a remit to promote health and prevent illness. Population health data is crucial to achieving the organisation’s 10-year goal of increasing physical activity and healthy eating, improving mental wellbeing, preventing harm from alcohol and preventing tobacco use [7] (although tobacco is not included in this study). These risk factor areas have been selected due to their major impact on the burden of disease in Australia [1].

The health promotion and illness prevention work of VicHealth is designed to complement the implementation of the Victorian Government Department of Health and Human Services (DHHS) Victorian Public Health and Wellbeing Plan [8]. This plan is in part delivered through Local Government Areas (LGAs) or councils. These local governments are part of Australia’s three-tier system of elected government: federal, state and local. There are 79 local governments in Victoria, 31 in the metropolitan area of Melbourne and the remainder are in the regional or rural areas of Victoria. Local government is responsible for planning and delivery of services including local building and planning control, human and community services, and health. In regard to health, local governments are responsible for the development of their Municipal Public Health and Wellbeing Plans (MPHWP) every 4 years. These are designed to be a local extension of the statewide Victorian Health and Wellbeing Plan.

By Act of the Victorian Parliament, MPHWPs must be informed by various evidence sources including population health data [9], therefore these plans require data from population health surveys to provide information about local public health needs. Since 2007, VicHealth has conducted population surveys in Victoria every 4 years to address this requirement, focusing on a range of behavioural risk factors that vary with each survey iteration to adapt to the needs of the state context. Known as the Community Indicators Victoria in 2007 and VicHealth Indicators (VHI) Survey in 2011, data from these population surveys have been critical to informing the development of local government plans, along with the DHHS Victorian Population Health Survey (VPHS). An examination of use of previous VHI Survey (2011) data found that it was amongst the top three of the most commonly cited sources of data informing MPHWPs [10], along with the Australian Bureau of Statistics (ABS) and VPHS. However, it is not known how many LGAs use VHI data and the degree to which this use varies according to the regional or socio-economic characteristics of LGAs.

The process of population data collection, analysis, and publication for use by policy makers or practitioners is complex. The World Alliance for Risk Factor Surveillance has identified three major challenges for risk factor surveillance: use of appropriate measures so that the most relevant data can be captured; use of appropriate sampling and data collection methodologies to enable production of reliable and robust data; and the third, which has been less well researched and reported, the use of health risk factor surveillance data by those who create health promotion policy and plans [11]. The VHI survey was designed to address these challenges, hence the primary objectives of this paper are to:

1. describe the methodology of the VicHealth Indicators survey
2. report on the prevalence of a range of health related behavioural and attitudinal risk factors for Victoria and their distribution according to sociodemographic characteristics; and
3. examine the use of these data in Victorian Municipal Public Health and Wellbeing Plans 2017–2021 according to local area characteristics.

In addition, to ensure that this paper provides a strong foundation for subsequent research utilising this large population data set, the STROBE checklist for reports of observational studies [12] has been used to structure the survey reporting component of this paper. The checklist requirements are designed to ensure accurate and complete reporting of observational cross-sectional studies such as the VHI survey.

## Methods

### Part 1 – VHI survey

#### Study design

The VHI was a cross-sectional survey using computer-assisted telephone interviews (CATI) that were applied to the adult population aged 18 years and over for the state of Victoria, Australia. The 15-min survey focused on a range of behavioural and attitudinal health risk factors. It was conducted from October to December 2015 using a dual-frame approach, that is, using landline and mobile phone numbers. The survey interviews were conducted by the Social Research Centre on behalf of VicHealth.

#### Setting

The Australian state of Victoria is located in the south-east corner of the country and according to the Australian census for 2016 [13] had the second highest population of all states with 5.9 million people. Females comprised 50.9% of the population, the median age was 37 years and the majority (70%) lived in the capital city of Melbourne. Most of the population were born in Australia (64.9%), followed by England (2.9%), India (2.9%), China (2.7%), New Zealand (1.6%) and Vietnam (1.4%), and 0.8% were Aboriginal or Torres Strait Islander people. After English (67.9%), the most common languages spoken at home were Mandarin (3.2%), Italian (1.9%), Greek (1.9%), Vietnamese (1.7%) and Arabic (1.3%).

In a survey of 133 cities conducted in 2017, Melbourne was ranked the 15th most expensive city in the world [14]. However, it is important to note that from 2011 to 2015, Melbourne was ranked as the world’s most livable city by the Economist Intelligence Unit’s (EIU) livability survey of 140 cities [15]. The state is governed by the Government of Victoria and is one of six States and two Territories in the federated Government of Australia. It is divided into 79 local government areas or municipalities.

#### Participants

The VHI survey was undertaken using a CATI approach and two random digit dialing (RDD) sample frames. One sample frame comprised randomly-generated landline telephone numbers for Victorian residents and the other randomly-generated mobile phone numbers that could be located anywhere in Australia. Both sample frames were purchased from the commercial sample provider Sample Pages [16].

The in-scope population for the survey was residents of Victorian households aged 18 years and older contactable via a telephone (either landline or mobile). The survey excluded non-Victorian residents, those whose postcode or location of residence could not be determined, non-private dwellings such as hotels and motels, hostels, boarding schools and boarding houses, hospitals, nursing and convalescent homes, prisons, reformatories and single quarters of military establishments, those under the age of 18 years, Victorian residents who were away for the duration of the fieldwork period, Victorian residents who declared they were too unwell or unable to do the survey, and those who spoke a language other than English that was not one of the top ten languages spoken in Victoria for which an interpreting service was provided.

Survey participants were recruited from every LGA in Victoria, with a sample size of approximately 300 in most LGAs, and a reduced sample size of 200 in the 10 least populous LGAs. This sampling approach was used to obtain sufficient numbers to allow LGA-level analysis. Given the differences in population sizes between LGAs (ranging from Queenscliffe [*n* = 3017] to Greater Geelong [*n* = 229,420]) [17], householders in Victoria’s least populous LGAs would have as high as 1 in 10 chance of being selected in a survey with 300 interviews in each LGA. This compared with other LGAs, where the chance of a household being selected may have been as low as 1 in 116. Therefore, the ten least populous LGAs had a reduced sample size of 200 to reduce the odds of selection. The sample size estimate was compiled from previous dual-frame surveys conducted by the Social Research Centre across a number of large-scale projects.

As the proportion of mobile-only residents had increased rapidly over the last decade (estimated to be 29.0% of adult Australians as at December 2014 [18]), it was necessary to conduct the survey using a dual-frame survey design incorporating both landline and mobile sampling frames. In order to adequately represent the mobile-only population segment, the target for mobile interviews was set at 35% of all interviews. Hence, it was anticipated that approximately 8063 of the 22,700 interviews would be conducted by mobile phone. The number of mobile phone connections is not evenly distributed across LGAs, as both mobile phone coverage (percentage of population with a mobile phone) and total number of residents per LGA varies. Accordingly, it was expected that the number of mobile phone interviews would also vary by LGA. To ensure that every LGA would have a mix of landline and mobile interviews, and thus each household within the LGA would have a chance of selection, a minimum quota of 30 landline interviews per LGA was set.

##### Landline sample

For the landline sample, a ‘best estimate’ of postcode was assigned to each record at the number generation and testing stage, based on information available about the geographic area serviced by each individual telephone exchange. The landline sample was generated at the LGA level.

All Victorian residential landline telephone numbers were considered in-scope. Accordingly, certain groups within the Victorian population were unable to be recruited into the landline sample for the survey. These groups included those living in facilities such as aged-care homes, prisons or hospitals, and homeless persons. Further, anyone who stated that they were unable to participate in a telephone survey, for health or other reasons, was excluded from the survey. To establish the landline sample list, all available numbers within each telephone exchange across Victoria were generated and tested to determine if they were working telephone numbers. Based on the location of the exchange that generated the telephone number, an initial LGA selection and postcode was allocated to each sample record to guide sample loading and ensure that sufficient sample records were generated. The final allocation of LGA was based on postcode and locality information provided by the respondent during the interview process. Prior to the survey, a primary approach letter was mailed to each landline sample member where a full address match could be found. The generated landline sample member file was compared to commercial lists to identify valid numbers. Next, a matching service was used to identify names, addresses and telephone number combinations which remained current. The reference database was the online version of the White Pages directory [19].

Respondent selection within a household for the landline component of the survey was undertaken using the ‘next birthday’ method for those aged 18 years or older to ensure random selection of adult participants.

##### Mobile sample

For the mobile phone sample, all mobile numbers were considered in-scope provided the person answering the phone lived in Victoria and was aged 18 or older. Phone numbers were generated and tested, based on the known mobile phone prefixes, to determine if they were legitimate mobile numbers. In Australia, randomly-generated mobile telephone numbers do not have geographic information attached to them, therefore many screening calls were potentially needed in order to identify Victorian residents. To increase the likelihood of reaching a Victorian respondent, a short message service (SMS) was sent to mobile sample members with the aim of informing the mobile owner about the survey and ascertaining whether they were a Victorian resident via return SMS. This also served to increase the proportion of mobile sample members who would answer a voice telephone call from a number who would otherwise remain ‘unknown’. There was no additional respondent selection for mobile phone survey participants. The person answering the phone was selected, if in-scope.

#### Variables

##### Indicator variables

The selection of indicator variables was guided by several key principles:

- Alignment to the imperative areas of VicHealth’s 10-year strategic plan
- Sensitivity to change across person, place and time
- Amenable to action at a range of jurisdiction levels including local government
- Complement and do not duplicate items in other population surveys, most notably the VPHS
- Items needed to be brief so that that could also be feasibly used in local program evaluations, thus allowing the population measure to act as a comparator for local evaluations
- Indicators were selected for their strong psychometric properties wherever possible
- Continuation of general wellbeing, life satisfaction and perceived safety items from previous VicHealth population surveys in 2007 and 2011

The selected indicator variables, the question source and psychometric properties are outlined below. The questionnaire is in Additional file 1.

***General wellbeing*** Three general wellbeing indicators were included in the survey, as they were longitudinal items used in two previous VicHealth population surveys. The first was subjective wellbeing, which was measured using the Australian Unity Personal Wellbeing Index [20]. The Index included ratings across seven domains: standard of living; health; achievements in life; community connection; personal relationships; safety; and future security. The average scores in all seven domains were combined into a Personal Wellbeing Index score and converted into a scale score ranging from 0 (completely dissatisfied) to 100 (completely satisfied). Four of the eight items (satisfaction with life as a whole, health, safety, feeling part of the community) were part of the core set of questions asked in every wave of the Household, Income and Labour Dynamics in Australia (HILDA) longitudinal cohort survey [21].

The Australian Unity Wellbeing Index has established Australian psychometric properties and normative Australian reference data. Typically, individual scores vary significantly, but are usually between 55 and 95 points. Personal wellbeing scores below 50 can be an indicator of depression [20].

***Satisfaction with life as a whole*** Satisfaction with life as a whole is the first item of the Australian Unity Personal Wellbeing Index [20]. Life satisfaction measures how people evaluate their life as a whole, rather than their current feelings.

***Perceptions of safety*** Two indicators were reported for perceptions of safety: (1) perception of safety while walking alone during the day, and (2) perception of safety while walking alone after dark. Each score was presented on a 5-point Likert Scale ranging from Very Safe to Very Unsafe. The question was first used in the ABS General Social Survey [22]. The perception of safety while walking alone after dark question has also been used in the VPHS as a measure of trust and social cohesion [23].

The base for this indicator comprised all survey participants, including those who selected “Don’t know”, refused response, or advised that the scenario wasn’t applicable to them. This approach mirrored the analysis undertaken by the ABS, who also use these survey items in the General Safety Survey.

***Mental wellbeing*** Five mental wellbeing indicators were reported. The resilience indicator reported a score derived from two items measured on a scale of 0–8, where 8 represents the highest possible level of resilience. The indicator was derived using the abbreviated Connor-Davidson Resilience Scale (CD-RISC 2) [24], a two-item measure with published psychometric properties. The 2015 VicHealth Indicators survey was the first time a resilience measure such as this had been used with the general population in Australia.

The next three mental wellbeing indicators related to social connection and people’s perception of their local neighbourhood, as there is an increasing amount of research demonstrating that neighbourhood cohesion impacts on mental health and wellbeing [25]. Each indicator was derived from a score on a single-item statement. The statements were: (1) people in this neighbourhood can be trusted, (2) this is a close-knit neighbourhood, (3) people around here are willing to help their neighbours. Each item was scored on a 5-point Likert scale ranging from Strongly Agree to Strongly Disagree. These items had previously been used in Australia in the HILDA survey (waves 6, 10, 14) [21] and first appeared in the Project on Human Development in Chicago Neighborhoods (wave 3) [26].

The fifth mental wellbeing indicator referred to the perception of gender equality in relationships. Gender equality influences the formation of attitudes which support violence against women. This is important in relation to health, as intimate partner violence is a risk factor for depression and anxiety in women [27]. Weak support for gender equality has been found to be associated with violence-supportive attitudes [28]. The indicator was based on the Gender Inequality in Relationships Scale [29] . Scores were derived from two items measured on 5-point Likert scales rated from Strongly Agree to Strongly Disagree, which were then combined and converted into scores out of 100. Scores on this indicator are divided into three categories, where low represented a score equal to or less than 70, medium represented a score of 80 or 90, and high represented a score of 100. The proportion of those with a low gender equality score was used as an indicator of gender equality for the VHI Survey. Respondents who gave a null answer (‘don’t know’ or ‘refused’) to both statements were excluded from the analysis, consistent with the scoring instructions for the scale.

***Physical activity*** Measurement of physical activity occurred in three ways: level of physical activity, type of physical activity, and whether that physical activity was undertaken through an organisation such as a sports club, or as a non-organised self-directed activity such as walking.

Level of physical activity was ascertained using a single item asking respondents about the number of days in a usual week during which they would accumulate 30 min or more of physical activity which was enough to raise breathing rate. The 30 min did not have to be continuous and could be completed over ten -minute increments throughout the day. This single-item measure has been shown to possess good criterion validity when compared with estimated physical activity levels based on accelerometer data [30, 31]. It was used in the 2015 VicHealth Indicators survey as a parsimonious approach to measurement of physical activity that could also be used as a field measure in evaluation projects. The sub-components of physical activity at the time for the survey were 0 days of 30 min of physical activity (defined as inactive), 1–3 days of 30 min of physical activity (defined as insufficiently active), and 4 days or more of 30 min of physical activity (defined as sufficiently active). These sub-classifications were based on the results of the Milton et al. study (2013), which indicated that 4 days or more of physical activity was a sufficient indication of adequate physical activity given that the measure did not account for physical activity associated with occupational activity or household chores.

There were three indicators relating to ‘organized physical activity’, that is, physical activity organised by a club, association or other organisation. The first indicator reported the percentage of individuals who usually partake in any organised physical activity, irrespective of the organiser. The second reported the percentage of individuals partaking in physical activity organised by a fitness, leisure or indoor sports centre, while the third indicator reported the percentage of individuals partaking in physical activity organised by a sports club or association. The three measures these indicators were derived from, were specifically developed for the VicHealth Indicators survey to provide unique information about the physical activity patterns of Victorians that were not available in any other surveys.

There were six indicators relating to ‘non-organised physical activity’. The first reported on the percentage of individuals partaking in any non-organised physical activity, for instance going for a run. Like the participation in organised physical activity indicator, the indicator for participation in non-organised physical activity was specifically developed for the VicHealth Indicators, and provided information not available from any other survey. The other five indicators report the percentage of individuals who nominated (1) walking, (2) jogging or running, or (3) cycling, as one of their main three types of non-organised physical activity, and whether they participate in non-organised activity alone or with someone else. These three activities (walking, jogging, cycling) were selected for reporting as they constitute the top 3 non-organised physical activities.

***Sedentary behaviour at work*** One indicator for sedentary behaviour at work was used. This indicator represented the average time individuals reported sitting at work on a usual day and was based on a single item. The item was a variation of the sitting at work question in the National Health Survey [5], with the reporting period changed from “in the last week” to “on a usual day”.

Only respondents aged 18–64 years who worked 35 or more hours a week were in scope for this indicator. This resulted in a sub-sample of 27.7% of all respondents. A further 154 respondents provided a null response (‘don’t know’ or ‘refused’) to this survey item and were excluded from the mean calculation. Three respondents gave a response of greater than 17 h, and were excluded, as 17 h represented a double shift and it was deemed unlikely that respondents would typically work more than a double shift.

***Healthy eating*** Four indicators for healthy eating were used: fruit, vegetable, take-away food and water consumption. An individual’s consumption of vegetables and fruit was expressed as the mean number of serves eaten per day. Both indicators referred to a single item that recorded individuals’ number of serves. A serve of vegetables was defined as half a cup of cooked vegetables or 1 cup of salad vegetables. Potato crisp and vegetable juice consumption did not count towards vegetable consumption. Those unable to report their vegetable consumption were excluded from the analysis (1.1%), likewise those who reported that they consumed more than 17 serves (double the recommended guidelines of 5 serves per day plus 3.5 serves of protein) of vegetables in a typical day were excluded (less than 0.1%) [32]. A serve of fruit was defined as one medium piece or two small pieces of fruit or one cup of diced fruit pieces. Fruit juice consumption did not count towards daily fruit consumption. For fruit consumption, 0.9% were unable to report their consumption, while less than 0.1% reported consuming more than ten serves of fruit in a typical day. Both groups were excluded from analysis.

The inclusion of consumption of fruit and vegetables in the survey reflected the importance of these measures as proxy indicators of a healthy diet [33].

As an indicator of an unhealthy diet, consumption of take-away meals or snacks was measured. This indicator captured the percentage of individuals eating take-away food or snacks – such as pizza, hamburgers, or hot chips - on three or more days per week. It was based on a single graduated frequency item, which was specifically developed for the 2015 VicHealth Indicators Survey. It complements the vegetable and fruit indicators by providing indirect information on the consumption of discretionary food. The qualifying examples of take-away and snack food were inserted following cognitive testing which showed the need for disambiguation.

Water consumption was measured using the average number of cups of water (250 ml) usually consumed by individuals. Those unable to report their water consumption were excluded from the analysis (0.6%), likewise those who reported that they consumed more than 30 cups (or 7.5 l) of water in a typical day were excluded (0.1%).

The water consumption item had previously been used in the New South Wales (NSW) Population Health Survey [34].

***Alcohol*** Three indicators regarding alcohol consumption were measured. The first indicator represented the percentage of individuals who reported consuming 5 or more standard drinks in a single sitting at least monthly. The second indicator represented the percentage of individuals consuming more than 11 standard drinks in a single sitting at least monthly. According to Australian alcohol guidelines [35], a standard drink is comprised of 10 g of alcohol which is equivalent 1 pot/middy of full strength beer, 1 small glass of wine or 30 ml of spirits. Each of the two indicators was based on a graduated frequency question, which is a common question design in alcohol consumption surveillance studies [36]. The cut-off selected as an indicator of alcohol short-term harm was based on the National Health and Medical Research Council’s Australian Guidelines to Reduce Health Risks from Drinking Alcohol 2009 [35]. Risk of short-term harm was defined as 5 or more standard alcoholic drinks in one session on at least one occasion each month, and very high risk was defined as 11 or more standard alcoholic drinks in one session on at least one occasion each month.

The third alcohol indicator referred to general attitudes towards alcohol, specifically individuals’ attitude towards drunkenness. This was measured by two similar items scored on a five-point Likert scale ranging from Strong Agree to Strongly Disagree. The indicator reflected the percentage of individuals who agreed or strongly agreed to the question “Do your FAMILY and FRIENDS agree or disagree that getting drunk every now and then is OK? “and “Do you PERSONALLY agree or disagree that getting drunk every now and then is OK?”, where getting drunk was defined as the point of losing balance. The question phrasing was a variation of the original phrasing of this item “‘getting drunk now and again is not a problem” as used in the Victorian School Students and Drug Use Supplementary Survey [37].

##### Indicator measures

Table 1 below outlines the indicator variables, the variable questions, the individual question scoring and the derived variable for population level scoring reported for use in Victorian policy and programs.

**Table 1:** Indicator, indicator question, response frame, and score processing

INDICATOR	QUESTION	INDIVIDUAL ITEM SCORING	POPULATION LEVEL SCORING	RE-TEST RELIABILITY COEFFICIENT
**General wellbeing**				
Subjective wellbeing [range 0–100]	Thinking about your own life and your personal circumstances, how satisfied are you with your life as a whole? Turning now to various areas of your life...How satisfied are you with your standard of living? … with your health? … with what you are currently achieving in life? … with your personal relationships? … with how safe you feel? … with feeling part of your community? … with your future security?	Scale from 0 to 10, where 0 is completely dissatisfied and 10 is completely satisfied, answered for each domain area.	Average scale score Average score of 8 domains is combined into a Personal Wellbeing Index score and converted into a scale maximum score with a range of 0 (completely dissatisfied) to 100 (completely satisfied).	Kappa = 0.84 [38]
Satisfaction with life as a whole [range 0–10]	Thinking about your own life and your personal circumstances, how satisfied are you with your life as a whole?	Scale 0–10, where 0 is completely dissatisfied and 10 is completely satisfied.	Average scale score	
Perceptions of safety – walking alone during day	Now a question about safety...How safe or unsafe do you feel when you are in the following situations?...Walking in your local area alone during the day How safe do you feel...? Walking in your local area alone after dark	Very safe; Safe; Neither safe nor unsafe; Unsafe; Very unsafe; Never alone in this situation	% of respondents who feel ‘safe’ or ‘very safe’ walking alone in their local area during the day.	
Perceptions of safety – walking alone after dark	Now a question about safety...How safe or unsafe do you feel when you are in the following situations?...Walking in your local area alone during the day How safe do you feel...? Walking in your local area alone after dark	Very safe; Safe; Neither safe nor unsafe; Unsafe; Very unsafe; Never alone in this situation	% of respondents who feel ‘safe’ or ‘very safe’ walking alone in their local area after dark.	
**Mental wellbeing**				
Resilience [range 0–8]	Able to adapt to change … Tend to bounce back after illness or hardship^a^ ^a^actual question text is proprietary to CD-RISC 2 questionnaire and thus cannot be reproduced here.	Scale 0–4 where: Not true at all = 0 Rarely true = 1 Sometimes true = 2 Often true = 3 True nearly all the time = 4 (Don’t know) (Refused)	Average scale score Scale score is sum of the two item scores for the two items on a scale of 0–8.	Intraclass Correlation = 0.86 [24] ^a^‘Adapt to change’ Kappa = 0.38^a^ ^a^‘Bounce back’ Kappa =0.33
Perceptions of neighbourhood - people are willing to help each other	Now some general questions about your neighbourhood: On a scale of 1 to 7, where 1 is strongly disagree and 7 is strongly agree, do you agree or disagree that …? ^a^(STATEMENTS) a. People around here are willing to help their neighbours b. This is a close-knit neighborhood c. People in this neighbourhood can be trusted IF RESPONDENT UNSURE ABOUT NEIGHBOURHOOD, SAY: It is whatever you think of as your neighbourhood – this can mean your local area	1. 1 - Strongly disagree 2. 2 3. 3 4. 4 5. 5 6. 6 7. 7 – Strongly agree 8. (Don’t know / not stated) 9. (Refused)	% of respondents who agree with statement 1 (score = 5|6|7).	^a^Kappa = 0.38
Perceptions of neighbourhood - this is a close-knit neighbourhood	Now some general questions about your neighbourhood: On a scale of 1 to 7, where 1 is strongly disagree and 7 is strongly agree, do you agree or disagree that …? ^a^(STATEMENTS) a. People around here are willing to help their neighbours b. This is a close-knit neighborhood c. People in this neighbourhood can be trusted IF RESPONDENT UNSURE ABOUT NEIGHBOURHOOD, SAY: It is whatever you think of as your neighbourhood – this can mean your local area	1. 1 - Strongly disagree 2. 2 3. 3 4. 4 5. 5 6. 6 7. 7 – Strongly agree 8. (Don’t know / not stated) 9. (Refused)	% of respondents who agree with statement 2 (score = 5|6|7).	^a^Kappa =0.38
Perceptions of neighbourhood - people can be trusted	Now some general questions about your neighbourhood: On a scale of 1 to 7, where 1 is strongly disagree and 7 is strongly agree, do you agree or disagree that …? ^a^(STATEMENTS) a. People around here are willing to help their neighbours b. This is a close-knit neighborhood c. People in this neighbourhood can be trusted IF RESPONDENT UNSURE ABOUT NEIGHBOURHOOD, SAY: It is whatever you think of as your neighbourhood – this can mean your local area	1. 1 - Strongly disagree 2. 2 3. 3 4. 4 5. 5 6. 6 7. 7 – Strongly agree 8. (Don’t know / not stated) 9. (Refused)	% of respondents who agree with statement 3 (score = 5|6|7).	^a^Kappa = 0.44
Low gender equity score	The statements I’m about to read out describe different attitudes that people have. Please tell me whether you strongly agree, somewhat agree, somewhat disagree or strongly disagree. a. Men should take control in relationships and be the head of the household b. Women prefer a man to be in charge of the relationship	5. Strongly agree; 4. Somewhat agree; 3. Neither agree nor disagree; 2. Somewhat disagree; 1. Strongly disagree; (Don’t Know / Can’t Say); (Refused)	% low gender equity Score for each question multiplied by 10, then both question scores summed. Low gender equity is score < 70.	
High gender equity score	The statements I’m about to read out describe different attitudes that people have. Please tell me whether you strongly agree, somewhat agree, somewhat disagree or strongly disagree. a. Men should take control in relationships and be the head of the household b. Women prefer a man to be in charge of the relationship	5. Strongly agree; 4. Somewhat agree; 3. Neither agree nor disagree; 2. Somewhat disagree; 1. Strongly disagree; (Don’t Know / Can’t Say); (Refused)	% high gender equity Score for each question multiplied by 10, then both question scores summed. High gender equity is score > 90.	
**Physical Activity**				
0 days per week 1–3 days per week 4+ days per week	In a usual week, on how many days do you do a total of 30 min or more of physical activity, which was enough to raise your breathing rate? This may include sport, exercise and brisk walking or cycling for recreation or to get to and from places, but should not include housework, gardening or physical activity that may be part of your job.	None; Number of days given (1–7); (Not applicable); (Don’t know) (Refused)	% of respondents selecting ‘None’. % of respondents reporting 1–3 days. % of respondents reporting 4+ days.	^a^Kappa = 0.53
*Organised physical activity*				
Participation in any organised physical activity	Is the [(name of sport/physical activity)] organised by a club, association or other organisation?	1. Yes 2. No 3. (Don’t know) 4. (Refused)	% answering ‘Yes’	^a^Kappa = 0.81
Organised by a fitness, leisure or indoor sports centre	What type of club, association or organisation organised the [<name of sport/physical activity>]?	1. Fitness, leisure or indoor sports centre	% participating in sport via a fitness, leisure or indoor sports centre	^a^Kappa =0.41
Organised by a sports club or association	What type of club, association or organisation organised the [<name of sport/physical activity>]?	2. Sports club or association	% participating in sport via a sports club or association	^a^Kappa = 0.78
*Non-organised physical activity*				
Participation in any non-organised physical activity	Is the [(name of sport/physical activity)] organised by a club, association or other organisation?	1. Yes 2. No 3. (Don’t know) 4. (Refused)	% answering ‘No’	^a^Kappa = 0.64
Activity type - walking	What are the three main types of physical activities that you USUALLY do?	Free response	% of respondents mentioning ‘Walking’ as one of their top three physical activity types AND declared it as a non-organised activity type.	
Activity type - jogging or running	What are the three main types of physical activities that you USUALLY do?	Free response	% of respondents mentioning ‘Jogging’ or ‘Running’ as one of their top three physical activity types AND declared it as a non-organised activity type.	
Activity type - cycling	What are the three main types of physical activities that you USUALLY do?	Free response	% of respondents mentioning ‘Cycling’ as one of their top three physical activity types AND declared it as a non-organised activity type.	
Participates alone	Who do you usually do the [<name of sport/physical activity>] with?	By yourself With friends/family Other (specify) (Don’t know) (Refused)	% of respondents selecting ‘By yourself’	
Participates with someone	Who do you usually do the [<name of sport/physical activity>] with?	By yourself With friends/family Other (specify) (Don’t know) (Refused)	% of respondents selecting ‘With friend/ family’ AND ‘Other’, where other is not a pet.	
*Sedentary behaviour at work*				
Time spent sitting on usual work day (hours: minutes)	The following question is about sitting at work, including meal and snack breaks and time spent sitting at a desk. How much time do you spend sitting at work on a usual work day?	Record time (hours/ minutes) per day; Did not sit at work; (Don’t know) (Refused)	% who spend 6 or more hours sitting in a typical work day	^a^Pearson correlation = 0.92
**Healthy Eating**				
Number of serves of vegetables per day	Now some questions about food. How many serves of vegetables do you USUALLY eat each day - a ‘serve’ is ½ cup of cooked vegetables or 1 cup of salad vegetables. NB: “Vegetables” includes potatoes, hot potato chips, but excludes potato crisps and excludes vegetable juice.	Record number of serves PER DAY; (Don’t know) (Refused)	Average number of serves per day	Interquartile range = 1.71 [39]
Number of serves of fruit per day	How many serves of fruit do you USUALLY eat each day - a ‘serve’ is 1 medium piece or 2 small pieces of fruit or 1 cup of diced pieces. NB: Excludes fruit juice	Record number of serves PER DAY; (Don’t know) (Refused)	Average number of serves per day	Interquartile range = 0.14 [39]
Eats take-away meals 3 or more days per week	How often do you eat take away meals and snacks that are bought from fast food or takeaway food outlets? Examples could be pizza, hamburgers, hot chips.	Most days (6–7 times per week); 3–5 times per week; 1–2 times per week; 2–3 times per month; Once per month; Less than once per month; Never; (Don’t know); (Refused)	% of respondents selecting ‘Most days’ or ‘3–5 times per week’.	^a^Kappa = 0.53
No water consumed per day	How many cups of water do you usually drink in a day? 1 cup = 250 ml or a household cup. 1 average 600 mL bottle of water = 2.5 cups.	Number of cups per day given or Number of litres per day given;	- Average cups per day	
Number of cups of water consumed per day	How many cups of water do you usually drink in a day? 1 cup = 250 ml or a household cup. 1 average 600 mL bottle of water = 2.5 cups.	Number of cups per day given or Number of litres per day given;	- Average cups per day	
**Alcohol**				
At risk of short-term harm each month	How often do you drink five or more standard drinks in a single session? A standard drink is equal to 1 pot of full strength beer, 1 small glass of wine or 1 pub-sized nip of spirits.	1. Every day 2. 5–6 days a week 3. 3–4 days a week 4. 1–2 days a week 5. 2–3 days a month 6. About 1 day a month 7. Less often 8. Never 9. (Don’t know) 10. (Refused)	Percentage of people drinking five or more standard drinks in a single session at least once a month.	
At very high risk of short-term harm each month	How often do you drink eleven or more standard drinks in a single session?	1. Every day 2. 5–6 days a week 3. 3–4 days a week 4. 1–2 days a week 5. 2–3 days a month 6. About 1 day a month 7. Less often 8. Never 9. (Don’t know) 10. (Refused)	Percentage of people drinking 11 or more standard drinks in a single session at least once a month.	
Alcohol culture - getting drunk occasionally is OK, perceived	Do you agree or disagree that your FAMILY AND FRIENDS think that getting drunk every now and then is okay? By getting drunk I mean drinking to the point of ‘losing balance’.	Strongly agree; Somewhat agree; (Neither agree nor disagree); Somewhat disagree; Strongly disagree; (Don’t Know / Can’t Say); (Refused)	Percentage of people agreeing or strongly agreeing with the question statement.	^a^Kappa = 0.26
Alcohol culture - getting drunk occasionally is OK, personal	Do you PERSONALLY agree or disagree that getting drunk every now and then is okay? By getting drunk I mean drinking to the point of ‘losing balance’.	Strongly agree; Somewhat agree; (Neither agree nor disagree); Somewhat disagree; Strongly disagree; (Don’t Know / Can’t Say); (Refused)	Percentage of people agreeing or strongly agreeing with the question statement.	^a^Kappa = 0.32

^a^Results from test-retest reliability analysis conducted as part of this study

##### Socio-demographic variables

The socio-demographic indicators questions were selected to match those in the VPHS survey wherever possible to allow for consistency of data reporting. It included questions related to gender, age, household structure, Aboriginal status, country of birth, language spoken at home, education, income and main daily activity, geographic region and the Index of Relative Socio-economic Disadvantage (IRSD) of the ABS Socio-Economic Indicator for Areas (SEIFA) [40]. The sexuality and disability socio-demographic indicators were derived from the ABS General Social Survey [22]. Socio-demographic indicators, indicator questions, and indicator categories are summarized in Table 2, the full questions and response options are in the VHI questionnaire (Additional file 1).

**Table 2:** Sociodemographic indicator, indicator question, and indicator categories

Sociodemographic indicator	Question	Categories
Gender	Now I have some questions to help us analyse the results. Just to confirm, what is your gender?	Male
		Female
		Other
Age	How old were you last birthday?	18–24 years
		25–34 years
		35–44 years
		45–54 years
		55–64 years
		65–74 years
		75+ years
Household structure	Which of these BEST describes your household?	Single person household
		Couple household
		Household with children
		*Single parent with dependent children*
		*Couple parent with dependent children*
		Share or group household
Aboriginal status	Are you of Aboriginal or Torres Strait Islander origin?	Aboriginal and/or Torres Strait Islander
		Non-Aboriginal
Sexuality	Which of the following options best describes how you think of yourself?	LGBTI
		Heterosexual
Country of birth	In which country were you born?	Australian born
		Country of English speaking background
		Country of non-English speaking background
Main language spoken at home	Do you speak a language other than English at home?	English
		Other
Education	What is the highest year of schooling you have completed? (If relevant) What is the highest post-school educational qualification that you have obtained?	Some high school or less
		Completed high school
		TAFE/Certificate/Diploma
		University
Self-reported disability	Do you have a disability, health condition or injury that has lasted, or is likely to last, 6 months or more which restricts your everyday activities?	Reported disability - under 65 years
		Reported disability - over 65 years
		No disability reported
Income	Which of the following ranges best describes your <personal / household’s > approximate income, from all sources, before tax is taken out, over the last 12 months?	Less than $20,000
		$20,000 – $39,999
		$40,000 – $59,999
		$60,000 – $79,999
		$80,000 – $99,999
		$100,000 or more
Main activity	Which of these best describes your current main activity?	Employed
		Unemployed
		Student
		Home duties
		Retired
Geographic region	Derived from postcode	Metropolitan
		*inner metro*
		*middle metro*
		*outer metro*
		Interface
		Regional city
		Large shire
		Small shire
Region	Derived from postcode	Melbourne
		Rest of Victoria
SEIFA (Index of relative social disadvantage)	Derived from postcode	Lowest quintile (least disadvantaged)
		2
		3
		4
		Highest quintile (most disadvantaged)

#### Bias

##### Sample bias

As described earlier, with the increase in the proportion of Australians residing in mobile-only households, estimated to be 27.3% as at June 2014 [18], it had become clear that most general community telephone surveys needed to sample persons via both landlines and mobile phones in order to reduce the coverage gap and reach a representative sample of the population. In particular, 40% of those aged 18–24 years old and 51% of those aged 25–34 years old were mobile-only [18], and therefore no longer able to be reached successfully via the landline frame.

##### Non-response bias

Procedures to address non-response bias included an extended call regime, where up to six calls were placed to establish contact with a given household, and a further nine calls (if needed) were placed to secure an interview with the selected household member. Call attempts were spread over different times and days of the week. No interviewing took place on public holidays. Interviews were conducted in English and, where an interpreter was required, this was available for the six most common other languages in Victoria, that is Italian, Greek, Mandarin, Cantonese, Vietnamese, and Arabic [13], as well as Spanish, Korean, Serbian and Croatian.

Where soft refusals occurred, that is where initial contact with the household was identified as a refusal and the reason provided was ‘just hung up’, ‘not interested’ or ‘too busy’, participants were contacted a second time to ascertain willingness to participate.

In addition, a free telephone call 1800 number operated by the research company throughout the fieldwork period was used to handle interview logistics and general enquires relating to the survey.

##### Response bias

Cognitive testing was undertaken prior to piloting of the survey to ascertain respondents’ (*n* = 10) comprehension of intended meaning, the ability to retrieve an accurate answer, the extent to which respondents were willing to provide an honest response, and the extent to which they felt they could provide an accurate answer given the response scale options provided. As a result, a number of changes were made to the survey prior to pilot testing, particularly to the physical activity module, where several items had been developed for the VHI Survey. Subsequent pilot testing confirmed the integrity of the survey.

The pilot test for the survey was conducted on September 17 (*n* = 17). A second pilot test was conducted on September 23 (*n* = 30). Modifications to the survey instrument were made following this initial pilot test with a view to reducing the average interview length for the main study.

A test-retest reliability study was also undertaken (*n* = 300) with an interval of approximately 1 week. For scaled survey items (e.g. agree / disagree scales), the majority of respondents provided the same answer or one category different in the reliability survey as for the main survey (approximately 9 out of 10 respondents for each survey item). In terms of the categorical survey items asked in the reliability study, at least 9 out of 10 responded in the same way for both surveys. In regard to the time (in minutes) given for time spent sitting at work in a usual work day, the mean time was similar between both the main and reliability surveys. There was no significant difference between the two mean times and almost all respondents responded similarly across the two surveys. Intra-class correlations for continuous variables and kappa statistics for categorical variables from the 7-day test-retest reliability study are provided in Table 1.

#### Study size

As discussed previously, LGAs had a sample size of 300, excluding the ten least populous LGAs which had a reduced sample size of 200 in the final sample design. There are 79 LGAs in Victoria, which resulted in an expected sample of 22,700. Table 3 provides details regarding the standard of error and relative standard area for population proportions of interest at the state, large LGA and small LGA levels. All relative standard errors were lower than 25% and were therefore considered not to be subject to high sampling error.

**Table 3:** Standard error and relative standard error for population proportions

Area Type	Population	Population proportion of interest					
		50%		25%		10%	
		SE	RSE	SE	RSE	SE	RSE
**State**	5,937,481	0.003	0.66%	0.003	1.15%	0.0006	6.59%
**Large LGA**	85096^a^	0.029	5.77%	0.025	10.0%	0.017	17.32%
**Small LGA**	6583^a^	0.029	5.77%	0.03	12.09%	0.02	20.94 %

^a^Average population used for large and small LGAs

#### Statistical methods

Mean scores and sample proportions were used to estimate indicator prevalence according to derived variable definitions reported in Table 1. To ensure complementarity and consistency with statistical methods used over time in other major population health surveys in Victoria [23], statistically significant differences between groups were deemed to exist when confidence intervals of estimates did not overlap. To determine statistical significance, indicator prevalence estimates for one group of the population (for example, females) were compared with the estimate for all survey respondents at the state level. Missing data was excluded from the analysis.

As indicator estimates are known to be associated with demographic characteristics, particularly age and gender, it was important to adjust for these using a weighting procedure to ensure unbiased estimates at the whole-of-state level.

The weighting approach used a population weight comprising age and gender structure within each LGA, and age and gender structure and telephone status at the Victorian state population level. Several weighting approaches were examined and considered for use in the analysis of the survey, including age and gender structure within each LGA; age and gender structure within each LGA plus telephone status; and age and gender structure within each LGA, plus telephone status, birthplace and education. However, following an evaluation of the impact of this approach on the effective base at the state level, the inclusion of birthplace and education was considered inappropriate for use in the VHI survey, as it introduced too much variance to the estimates, thereby degrading their quality.

#### Ethics

The survey was approved by the Australian National University’s Human Research Ethics Committee (Approval number 2015/556).

### Part 2 – audit of VHI survey data citations in Municipal Public Health and Wellbeing Plans 2017–2021

Two products were prepared to enable dissemination of local-level results to LGAs. LGA profiles were prepared that summarized means or sample proportions for each indicator, as well as comparison to state level [41]. An online data portal was also prepared called Explore Your Data which enabled the user to select their LGA and explore the socio-demographic breakdown for each variable within the LGA, provided that sample sizes were adequate to allow comparisons [42].

Information about the LGA profiles and Explore Your Data were shared via local council mayors, local council CEOs, the Municipal Association of Victoria (MAV) website links and e-newsletter network, as well as local radio and newspaper press releases and interviews.

The audit of the MPHWPs was conducted between the 1st of November 2017 and the 28th of February 2018. The audit included a frequency count using three search terms; “VicHealth”, “Vic Health” and “Victorian Health Promotion Foundation” to capture any reference to the 2015 VHI survey. This micro-level data collection approach extracted every reference to the 2015 VHI and facilitated a robust content analysis. The 2017–2021 MPHWPs were obtained from 77 LGAs through either the MAV website [43], individual council websites or directly from council contacts. Two MPHWPs were not complete at the time of review.

The association between number of citations and geographic region type (i.e. inner metro, middle metro, outer metro etc.), and number of citations and LGA SEIFA were analysed using Welch’s ANOVA to conduct group comparisons without assuming equal variances [44].

## Results

### Part 1- VHI survey

#### Participants

The number of individuals at each stage of the study, from numbers potentially eligible through to those included in the study and analysed, is outlined below for the sample as a whole and for the landline and mobile frames:

- Completed interviews: Interviews accounted for 21.5% of final call outcomes across the landline frame and 3.9% of final call outcomes across the mobile frame;
- Eligible, non-interviews: Outcomes coded as household refusals were relatively rare when calling mobiles (0.7%) compared to when calling households (9.8%) and there was a lower proportion of unanswered final call outcomes via the mobile frame (15.7%) compared with 20.1% for the landline frame; and
- Unknown eligibility, non-interviews: A much higher proportion of answering devices were encountered via the mobile frame (14.4%) relative to the landline frame (2.8%). Similarly, a higher proportion of no answer was found within the mobile frame (32.9% compared with 24.2% in the landline frame).
- Not eligible: A much higher proportion of non-eligible sample members (e.g. not Victorian residents, not aged 18 years and over) were encountered via the mobile frame (29.6%) compared with the geographically targeted landline frame (0.2%).

The high proportion of ineligible numbers in the mobile phone frame is reflected in the high ratio of records used per interview for the mobile phone frame (25.9:1) compared with the landline frame (4.6:1) and is due to the unknown location associated with the mobile phone frame.

The final sample was 22,819. Table 4 outlines the original sample frame and the final response rate. The response rate used for this report is AAPOR Response Rate 3 (RR3) (American Association of Public Opinion Research, 2011). This relies on estimating the proportion of cases of unknown eligibility that may have been eligible for the survey and including this estimate in the denominator for the calculation of the survey response rate. The formula for Response Rate 3 is:

$$ \mathrm{RR}3=\frac{\mathrm{I}}{\left(\mathrm{I}+\mathrm{P}\right)+\left(\mathrm{R}+\mathrm{NC}+\mathrm{O}\right)+\mathrm{e}\left(\mathrm{UH}+\mathrm{UO}\right)} $$

**Table 4:** Final call disposition

	Total		Landline frame		Mobile frame	
	N	%	N	%	N	%
	277,832	100	68,429	100	209,403	100
***Interview (Category 1)***						
Complete	22,819	8.2%	14,737	21.5%	8082	3.9%
***Eligible, non-interview (Category 2)***						
Refusal and breakoff	403	0.1%	196	0.3%	207	0.1%
Known respondent refusal	7645	2.8%	2205	3.2%	5440	2.6%
Respondent never available	2220	0.8%	1392	2.0%	828	0.4%
Other, non-refusals	251	0.1%	173	0.3%	78	0.0%
Physically or mentally unable/incompetent	2454	0.9%	1951	2.9%	503	0.2%
Language problem	1154	0.4%	302	0.4%	852	0.4%
Location/Activity not allowing interview	13	0.0%	0	0.0%	13	0.0%
***Unknown eligibility, non-interview (Category 3)***						
Always busy	343	0.1%	82	0.1%	261	0.1%
No answer	85,560	30.8%	16,562	24.2%	68,998	32.9%
Answering machine	15,663	5.6%	9853	14.4%	5810	2.8%
Call blocking	1134	0.4%	146	0.2%	988	0.5%
Housing unit, unknown if eligible respondent	43,376	15.6%	8475	12.4%	34,901	16.7%
Other	673	0.2%	532	0.8%	141	0.1%
***Not eligible (Category 4)***						
Fax/data line	3763	1.4%	3334	4.9%	429	0.2%
Non-working/disconnect	20,190	7.3%	2851	4.2%	17,339	8.3%
Non-residence	7782	2.8%	5288	7.7%	2494	1.2%
No eligible respondent	62,152	22.4%	127	0.2%	62,025	29.6%
Quota filled	237	0.1%	223	0.3%	14	0.0%

*I* Interviews, *P* Partial interviews, *R* Refusals, *NC* Non-contacts, *O* Other, *E* Estimate of the proportion of unknown outcomes likely to have been in-scope, *UH* Unknown, if household / occupied *UO* Unknown other

Where:

The e value for this survey is the default value calculated by the AAPOR on-line Response Rate Calculator 6 [45]. In this case 0.282. This was calculated as follows:

$$ \mathrm{e}=\frac{\left(\mathrm{Interviews}+\mathrm{Partial}\ \mathrm{completes}\right)+\left(\mathrm{Eligible}\ \mathrm{non}-\mathrm{interviews}\right)}{\left(\mathrm{Interviews}+\mathrm{Partial}\ \mathrm{completes}\right)+\left(\mathrm{Eligible}\ \mathrm{non}-\mathrm{interviews}\right)+\left(\mathrm{Not}\ \mathrm{e}\mathrm{ligible}\right)} $$

On this basis, the overall response rate for the survey was 29.1%, response rate for the landline frame was 33.7% and the response rate for the mobile frame was 23.7% (see Table 5).

**Table 5:** Calculation of response rates

	Total sample	Landline	Mobile phone
***Total phone numbers used***	***277,832***	***68,429***	***209,403***
I=Complete Interviews (1.1)	22,819	14,737	8082
R = Refusal and break off (2.1)	8048	2401	5647
NC=Non-contact (2.2)	2220	1392	828
O=Other (2.0, 2.3)	3872	2426	1446
e	0.282	0.639	0.163
UH=Unknown Household (3.1)	102,700	26,643	76,057
UO=Unknown other (3.2–3.9)	44,049	9007	35,042
***Response Rate 3***			
I/((I + P) + (R + NC + O) + e (UH + UO))	29.1%	33.7%	23.7%

#### Descriptive data

Analysis of the achieved respondent profile by sample frame reveals some differences between the sample frames. As can be seen in Table 6, the mobile sample frame improves the representation of males, younger persons and those born overseas compared to benchmarking data from the ABS [46]. It is important to note that the proportion of LGBTI respondents in the sample was 3.9% (*n* = 894), however this is not included in Table 6 as there were no population benchmarks available for comparison.

**Table 6:** Characteristics of study participants by sample frame

		Sample		Landline	Mobile	Victorian population %
		N	%	%	%	%
	***Sub-category)***	***22,819***	***100*** ***(n = 22,819)***	***100*** ***(n = 14,737)***	***100*** ***(n = 8082)***	***–***
**Gender**	Male	9351	41	36	49	49
	Female	13,422	59	63	51	51
**Age group**	18–24 years	1218	5	2	11	13
	25–34 years	1969	9	3	19	20
	35–44 years	2631	12	8	17	18
	45–54 years	3698	16	15	19	17
	55–64 years	4841	21	23	18	14
	65–74 years	4883	21	27	12	10
	75 + years	3523	15	22	3	9
**Location**	Capital city	9903	43	26	75	75
	Rest of state	12,916	57	74	25	25
**Country of Birth**	Australian born	17,553	77	83	66	67
	Overseas born	5266	23	17	34	33
**Indigenous status**	ATSI^a^	190	1	1	1	1
	Non-ATSI^a^	22,532	99	99	99	99
**Educational attainment**	Bachelor degree	6654	31	25	41	25
	No Bachelor degree	6857	32	32	32	75

^a^Aboriginal and/or Torres Strait Islander

Key descriptive statistics for each of the outcome variables are outlined in Table 7.

**Table 7:** Summary of measures and associated key descriptive results

Indicator	Sample (N)	Out of scope	Missing	Lowest	Highest	Mean	Median	Mode	Standard Deviation
Subjective wellbeing [range 0–100]	21,472	0	1347	0	100	78.81	80	–	12.52
Satisfaction with life as a whole	22,697	0	122	0	10	7.92	8	–	1.67
Perceptions of safety – walking alone during day	22,819	0	68	1	3	1.09	1	1	0.36
Perceptions of safety – walking alone after dark	22,819	0	131	1	3	1.61	1	1	0.75
Resilience [range 0–8]	22,164	0	655	0	8	6.56	7	–	1.58
Perceptions of neighbourhood - people are willing to help each other	22,819	0	422	1	2	1.17	1	1	0.38
Perceptions of neighbourhood - this is a close-knit neighbourhood	22,819	0	464	1	2	1.28	1	1	0.45
Perceptions of neighbourhood - people can be trusted	22,819	0	635	1	2	1.19	1	1	0.39
Physical activity 0 days per week	22,819	0	116	–	–	–	–	–	–
Physical activity 1 to 3 days per week	22,819	0	116	–	–	–	–	–	–
Physical activity 4 or more days per week	22,819	0	116	–	–	–	–	–	–
Participation in any organised physical activity	22,819	0	32	1	2	1.73	2	2	0.44
Organised by a fitness, leisure or indoor sports centre	22,819	0	26	1	2	1.93	2	2	0.25
Organised by a sports club or association	22,819	0	26	1	2	1.9	2	2	0.3
Participation in any non-organised physical activity	22,819	0	32	1	2	1.32	1	1	0.47
Activity type - walking	22,819	0	3	1	2	1.45	1	1	0.5
Activity type - cycling	22,819	0	1	1	2	1.9	2	2	0.3
Activity type - jogging	22,819	0	0	1	2	1.92	2	2	0.27
Participates alone	22,819	0	17	1	2	1.48	1	1	0.5
Participates with someone	22,819	0	17	1	2	1.72	2	2	0.45
Time spent sitting on usual work day (hours: minutes)	6311	12,031	154	0	16:00	4:15	4:00	–	3:06
Number of serves of vegetables per day	22,559	0	260	0	16	2.42	2	–	1.55
Number of serves of fruit per day	22,621	0	198	0	10	1.66	1.05	–	1.12
Eats take-away meals 3 or more days per week	22,819	0	65	1	2	1.94	2	2	0.23
Number of cups of water consumed per day	22,693	0	126	0	30	4.86	4	–	3.22
At risk of short-term harm each month	22,819	0	43	1	2	1.77	2	2	0.42
At very high risk of short-term harm each month	22,819	0	29	1	2	1.94	2	2	0.24
Alcohol culture - getting drunk occasionally is OK (perceived)	22,819	0	762	1	2	1.78	2	2	0.41
Alcohol culture - getting drunk occasionally is OK (personal)	22,819	0	176	1	2	1.8	2	2	0.4
Gender equity category	22,819	0	213	1	3	2.06	2	3	0.82

#### Prevalence of behavioural and attitudinal risk factor indicators

The overall population prevalence of indicators referred to in Table 7 is provided in Additional file 2 and these are presented according to the following socio-demographics: gender (female/male), age group, geographic location of residence and socioeconomic status (SEIFA). Significant differences in prevalence rates between socio-demographic sub-populations and the total population are summarized below. The significant differences are referred to here as more or less favourable relative to the state prevalence estimate, given that it can be preferable to have a higher or lower score depending on the nature of the measure.

##### Gender

The prevalence of risk factors for females was more favourable than the state estimate for subjective wellbeing, gender equity attitudes, walking, fruit and vegetable consumption and both indicators of short term-harm from alcohol. Females’ scores were less favourable than the state estimate for perception of safety indicators (where at night it was half that of males), physical activity on 4 or more days, sports club involvement, participating alone in a non-organised sport, and sedentary behaviour (which was 30 min more on average than males).

The indicators that were more favourable than the state estimate for males were perceptions of safety, participation in sports clubs, running, cycling, and participation in non-organised activity alone. Prevalence of the following indicators for males were less favourable than the state estimate: subjective wellbeing; low support for gender equity (where the rate was almost double that of females); walking, fruit and vegetable consumption, and take-away food consumption (which was double that of females). Furthermore, most alcohol indicators were less favourable for males than the state estimate, with risk of short-term harm twice that of females, and very high risk of short-term harm three times that of females.

##### Age group

Regarding age, for younger age groups (18-34 years), the prevalence of the following indicators were significantly less favourable than the state estimate: resilience, all neighborhood indicators, gender equity, walking, consumption of take-away food, risk and very high risk of short term harm from alcohol, and alcohol culture (particularly the youngest age group of 18–24 year olds). For those aged 65 and over, prevalence rates were significantly more favourable than the state estimate for subjective wellbeing, life satisfaction, neighborhood connection, fruit and take-away food consumption and all alcohol indicators. Those aged 45 and over tended to have more favourable scores for resilience, gender equity, walking and take-away food consumption. Interestingly, there is a mid-life dip in subjective well-being and life satisfaction for those aged 35–54, where the prevalence was significantly lower than the state estimate, whereas resilience appeared to increase with age until 75 years and over where it declined slightly.

##### Geographic location

Considering geography, those living in rural areas, particularly shires, had significantly more favourable levels of subjective wellbeing, life satisfaction, perceptions of safety, resilience (regional cities as well), gender equity attitudes, neighborhood connection, participation in sports clubs, walking, sedentary behaviour, and vegetable and take-away food consumption. In contrast, outer metropolitan, and to some extent interface communities, which are located in between outer metropolitan and rural areas, tended to score less favourably on many indicators including perceived safety, resilience, neighborhood connectedness, gender equity, physical activity, participation in organized sport including sports clubs, cycling, and vegetable consumption. The only favourable score for this geographic segment was risk of short-term harm from alcohol, where outer metro was the only region with a more favourable result than the state average. The inner metropolitan area showed different characteristics, scoring significantly less favourably for alcohol harm, sedentary behaviour, takeaway food consumption and neighbourhood connectedness. Whereas this geographic segment had significantly higher prevalence rates than the state estimate for jogging, cycling, and going to a fitness center, and were the only region to score significantly more favourably than the state estimate for participating in physical activity on 4 days or more a week.

##### Socio-economic status

Regarding the variation in prevalence of indicators according to socio-economic status, there was a clear socio-economic gradient evident for most indicators, with significantly less favourable scores for those experiencing most disadvantage, and those with least disadvantage scoring significantly more favourably than the state estimate. However, a reversed gradient result was noted for two indicators, sedentary behaviour and risk of short-term harm from alcohol, where those experiencing most disadvantage scored significantly more favourably than the state estimate, and those experiencing least disadvantage scored significantly less favourably. The sedentary behavior may be a reflection of the higher blue-collar workforce participation in this demographic, and the lower risk of short-term harm from alcohol consumption finding is a pattern observed in other population studies [47]. The only indicators for which a socioeconomic status trend was not observed were: close knit neighborhood, fruit consumption and the perception that family and friends think getting drunk is OK.

### Part 2 - Audit of VHI survey data citations in Municipal Public Health and Wellbeing Plans 2017–2021

**Fig. 1:**
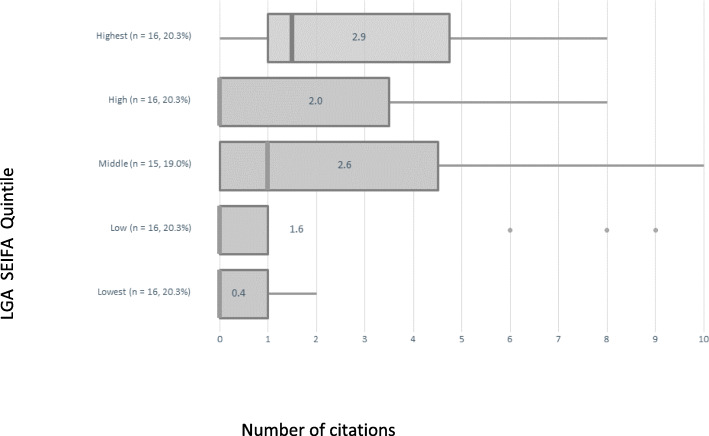
Number of citations of VHI data in LGA MPHWPs, according to LGA SEIFA (IRSD)

The audit of the 2017–2021 MPHWPs revealed that 38 out of 77 (49%) local governments directly cited VHI survey data in their plans. The VHI survey was referenced 145 times across the 38 plans. Citations did not differ between metropolitan and rural areas. However, the number of citations of VHI was significantly higher in the high SEIFA local government areas relative to the mean, and significantly lower in the low SEIFA local government areas relative to the mean (Fig. 1).

## Discussion

This paper has described in detail the methodology of the VicHealth Indicators survey and the prevalence of a range of behavioural risk factors that were identified through the survey. It is pleasing to see that the data has been considered useful by local Victorian policy makers and practitioners, with almost 50% of the Municipal Public Health and Wellbeing Plans 2017–2021 citing the survey data as a source of evidence to guide the development of Plans. In addition, it is the first study to examine variation in use of a data set for the development of health policy in Australia at the local government level according to geographic region type and area SEIFA.

The VHI 2015 data has provided a robust picture of behavioural and attitudinal risk factors for health. The sample size allows for confidence in the strength of the population estimates even when risks of bias are considered.

As the measures used in VHI and the way they were reported were designed to ensure that measures were brief enough to be used in project evaluations, and selected to complement other surveys, it is not possible to directly compare many outcomes to other Victorian or national survey outcomes. However, where comparisons are possible, and data are available for a similar period (2014–16), they are summarized here. For subjective wellbeing, the result for Victoria (77.3) was above the national average for Australia (76.1) [48]. The Victorian result for life satisfaction (7.8) was higher than Australian score of 7.6 [49] and the average international score of 7.3 [50]. For perceived safety walking in the local area at night, the result reported here for Victoria (55.1%) was higher than the national figure of 53.2% [49]. Comparable statistics are also available at a national level for risk (26%) and very high risk (15%) of short-term harm from alcohol [51], compared to the results reported here for Victoria of 29 and 9% respectively, although it is important to note that the national survey included younger respondents from 12 years of age and older. Whilst measures and methods of reporting were designed to differ from the VPHS, there are consistencies in the results, such as the majority of the population not meeting guidelines for vegetable consumption and less than half the population (VHI 41.3%, VPHS 41.4%) being defined as sufficiently active [23].

Prevalence estimates for 2017 have since become available for the proportion of Victorians meeting fruit and vegetable consumption guidelines, physical activity guidelines, alcohol injury risk and life satisfaction [8]. Results indicate that there has been no significant change in these indicators, apart from physical activity which has shown a significant increase of 3.9% in the proportion meeting national guidelines. Therefore, results reported in this survey are likely to still be relevant indicators of areas that require targeted action. Most notable overall is the average vegetable intake being 2.2 serves per day, far below the national guidelines of 5 serves per day, and only 4 in 10 Victorians being sufficiently active. This is particularly important given the link between these indicators and obesity, and over half the Victorian population being pre-obese or obese (54.5%) [8]. Other key areas for targeted action are low levels of neighborhood connection in younger adults, the high proportion of young males at risk of short-term harm from alcohol, low levels of support for gender equity among young males, and low levels of perceived safety amongst women when walking alone after dark.

Whilst there is clear variability in the prevalence of indicators according to age and gender, there is a consistent pattern of less favourable results for people living in the outer metropolitan area of Melbourne and to some extent those living in interface regions on the edge of the metropolitan area. It is important to note that the socio-demographic profile of regions in Victoria can vary substantially [13]. This may be a factor in the regional variability of indicators observed in this study. Interestingly, whilst there was considerable variation in indicator prevalence across geographic regions, the use of VHI 2015 data in MPHWP’s did not vary significantly between geographic region types.

The most striking finding is the socio-economic gradient that exists for most risk factors. This is consistent with the clear socio-economic gradient in risk factor contribution to disease burden [1], in that the main behavioural risk factors examined here increase in prevalence as SES declines, and their impact in terms of disease burden increases as well. Furthermore, there was a significant variation in use of VHI 2015 data in MPHWPs according to the SEIFA of the LGA, with lower SEIFA LGAs displaying significantly lower than average citations of VHI 2015, and high SEIFA areas displayed significantly higher than average. This is compelling evidence of the need for action to address health inequities to ensure that all Victorians can experience good health. This is particularly important as the results in this study indicate that low SES Victorians are potentially doubly disadvantaged, not only by their socio-economic status, but also through significantly lower utilisation of available population health data in local public health and wellbeing plans. It is important to note that all but one of the lowest SEIFA LGAs are rural shires, and average rural council budgets are one third of the average metropolitan council budget [38]. Hence, lack of public health staffing for the development of MPHWPs may be a factor in contributing to low rates of data use. In the future, greater use of this type of population health data in MPHWPs could be enabled through the use of new techniques that continue to emerge from the growing field of implementation science [52].

This study has a number of limitations. The use of landline and mobile phone for a CATI surveys can present some issues with bias. The primary approach for survey participation differs; landline using a posted letter whilst mobile using a short message service (SMS) to establish Victorian residence. In addition, the landline sample was less representative of the Victorian population, whereas the mobile phone sample was very comparable to the Victorian population in terms of key demographic breakdown. The overall sample under-represented males, young people aged 18–34, individuals born outside of Australia, those with an education level less than a university degree and those living in the capital city, the latter due to stratification of the sample frame according to LGA. Whilst weighting accounted for age, gender and telephone status, it did not weight for educational status or country of birth for reasons previously discussed, and this may have led to an underestimate of prevalence rates in the most disadvantaged SEIFA quintiles.

Although new items underwent cognitive testing and test-re-test reliability testing, their psychometric properties and performance in a population survey is unknown. In some instances, there was no benchmark to indicate what an acceptable level would be, unlike the subjective wellbeing measure [20], or where there are national guidelines such as amount of daily fruit and vegetable consumption [32]. Therefore, indicators such as resilience, neighborhood connectedness, gender equity and takeaway food consumption can only describe prevalence rather than the degree to which the population is reporting acceptable levels of the indicator.

Regarding the generalizability of findings, the indicators used for the first time in a survey of this size, such as the physical activity measure, resilience and alcohol culture questions, and establishment of their psychometric properties, will hopefully instill confidence in using these measures in population surveys in the future. In addition, a premise for selection of these measures was brevity so they could be used in evaluations in the future; this survey has established this to be a viable proposition. Generalizability of the findings to the whole Victorian population is a strength of this study as it is a large representative sample of Victorians. There are potential biases due to limitations of the representativeness of the sample, but these are substantially overcome by the weighting approach used.

## Conclusions

This paper has addressed the three key challenges identified by experts who have examined the importance and effectiveness of health risk surveillance for health promotion and public health [11]. These are: use of appropriate measures so that the most relevant data can be captured; use of appropriate sampling and data collection methodologies to enable production of reliable and robust data; and the third, which has been less well researched and reported, the use of health risk factor surveillance data by those who create health promotion policy and plans. The VHI program of work has met all three of these challenges and thus strengthened health promotion and illness prevention work in Victoria and provided insights into future action needed.

## Supplementary information

**Additional file 1.** VicHealth Indicators survey questionnaire. Listing of all survey questions.

**Additional file 2: Table S1.** Means or percent prevalences (with 95% CIs) of indicators according to gender, age, geographic region and SEIFA and comparison to State estimates. Table of estimates and significant differences between sub-population and whole of state estimate.

## Data Availability

The de-identified dataset used and analysed during this current study is available from the corresponding author on reasonable request. The de-identified dataset is anticipated to be available from the Department of Health and Human Services Open Data Site from December 2020 at http://data-dhs.opendata.arcgis.com/.

## Abbreviations

AAPOR: American Association of Public Opinion Research
ABS: Australian Bureau of Statistics
AIHW: Australian Institute of Health and Welfare
BRFSS: Behavioral Risk Factor Surveillance System
CATI: Computer Assisted Telephone Interview
CI: Confidence Interval
DHHS: Department of Health and Human Services
EIU: Economist Intelligence Unit
HILDA: Household, Income and Labour Dynamics in Australia
IRSD: Index of Relative Socio-economic Disadvantage
LGA: Local Government Area
MAV: Municipal Association of Victoria
MPHWP: Municipal Public Health and Wellbeing Plan
SEIFA: Socio-economic Indicator for Areas
SMS: Short Message Service
STEPS: STEPwise approach to Surveillance
VicHealth: Victorian Health Promotion Foundation
VHI: VicHealth Indicators
VPHS: Victorian Population Health Survey
WHO: World Health Organisation

## References

[1] Australian Institute of Health and Welfare (2019). Australian Burden of Disease Study: Impact and causes of illness and death in Australia 2015.

[2] G. B. D. Risk Factor Collaborators (2018). Global, regional, and national comparative risk assessment of 84 behavioural, environmental and occupational, and metabolic risks or clusters of risks for 195 countries and territories, 1990-2017: a systematic analysis for the global burden of disease study 2017. Lancet..

[3] Pierannunzi C, Hu SS, Balluz L (2013). A systematic review of publications assessing reliability and validity of the behavioral risk factor surveillance system (BRFSS), 2004–2011. BMC Med Res Methodol.

[4] Riley L, Guthold R, Cowan M, Savin S, Bhatti L, Armstrong T (2016). The World Health Organization STEPwise approach to noncommunicable disease risk-factor surveillance: methods, challenges, and opportunities. Am J Public Health.

[5] Australian Bureau of Statistics. National Health Survey, First Results, Australia 2014–15. 2015. https://www.ausstats.abs.gov.au/ausstats/subscriber.nsf/0/CDA852A349B4CEE6CA257F150009FC53/$File/national%20health%20survey%20first%20results.%202014-15.pdf. Accessed 18 Nov 2019.

[6] Portnoy B, Craddock Lee SJ, Kincheloe J, Breen N, Olson JL, McCormally J (2014). Independent state health surveys: responding to the need for local population health data. JPHMP..

[7] VicHealth (2019). VicHealth Action Agenda for Health Promotion 2019-2023.

[8] Department of Health and Human Services (2019). Victorian population health survey 2017.

[9] Parliament of Victoria (2008). Public Health and Wellbeing Act 2008.

[10] Browne GR, Davern M, Giles-Corti B (2017). What evidence is being used to inform municipal strategic planning for health and wellbeing? Victoria, Australia, a case study. Evid Policy.

[11] Campostrini S, McQueen D, Taylor A, Daly A (2015). World Alliance for risk factor surveillance White paper on surveillance and Health promotion. AIMS Public Health.

[12] von Elm E, Altman DG, Egger M, Pocock SJ, Gotzsche PC, Vandenbroucke JP (2014). The strengthening the reporting of observational studies in epidemiology (STROBE) statement: guidelines for reporting observational studies. Ann Intern Med.

[13] Australian Bureau of Statistics (2016). 2016 Census QuickStats.

[14] Economist Intelligence Unit (2015). Worldwide Cost of Living (WCOL) Survey.

[15] Economist Intelligence Unit (2015). 2015 Global Livability Ranking.

[16] Magenta Linas Software Pty Ltd (2015). Sample Pages.

[17] Department of Environment L (2016). Water and Planning,. Victorian Population Bulletin 2016.

[18] Australian Communications and Media Authority (2015). Snapshot: Australians get mobile: using mobile devices for voice, messaging and internet access.

[19] Sensis Pty Ltd (2015). White Pages.

[20] Cummins RA, Eckersley R, Pallant J, Van Vugt J, Misajon R (2003). Developing a national index of subjective wellbeing: the Australian Unity wellbeing index. Soc Indic Res.

[21] Watson N, Wooden M (2010). The HILDA survey: Progress and future developments. Aust Econ Rev.

[22] Australian Bureau of Statistics (2015). General Social Survey (GSS) March 2014 to June 2014 Household survey questionnaire.

[23] Department of Health & Human Services (2016). Victorian Population Health Survey 2014: Modifiable risk factors contributing to chronic disease.

[24] Vaishnavi S, Connor K, Davidson JRT (2007). An abbreviated version of the Connor-Davidson resilience scale (CD-RISC), the CD-RISC2: psychometric properties and applications in psychopharmacological trials. Psychiatry Res.

[25] Elliott J, Gale CR, Parsons S, Kuh D (2014). Neighbourhood cohesion and mental wellbeing among older adults: a mixed methods approach. Soc Sci Med.

[26] Earls FJ, Brooks-Gunn J, Raudenbush SW, Sampson RJ (2007). Project on human development in Chicago neighborhoods (PHDCN): community involvement and collective efficacy, wave 3, 2000–2002.

[27] Devries KM, Mak JY, Bacchus LJ, Child JC, Falder G, Petzold M (2013). Intimate partner violence and incident depressive symptoms and suicide attempts: a systematic review of longitudinal studies. PLoS Med.

[28] Heise LL, Kotsadam A (2015). Cross-national and multilevel correlates of partner violence: an analysis of data from population-based surveys. Lancet Glob Health.

[29] Webster K, Diemer K, Honey N, Mannix S, Mickle J, Morgan J (2018). Methodology report: Survey redevelopment and implementation of the 2017 National Community Attitudes towards Violence against Women Survey (NCAS) Sydney: ANROWS.

[30] Milton K, Clemes S, Bull F (2013). Can a single question provide an accurate measure of physical activity?. Br J Sports Med.

[31] O'Halloran P, Kingsley M, Nicholson M, Staley K, Randle E, Wright A (2020). Validity of the single item measure to assess change in physical activity. PLoS One.

[32] National Health and Medical Research Council (2013). Australian Dietary Guidelines.

[33] Australian Institute of Health and Welfare (2007). Australian diet quality index project.

[34] NSW Health (2014). NSW population Health survey 2014 - questionnaire Sydney: NSW Health.

[35] National Health and Medical Research Council (2009). Australian guidelines to reduce health risks from drinking alcohol.

[36] Livingston M (2015). Understanding recent trends in Australian alcohol consumption. Canberra.

[37] Department of Health and Human Services (2011). School students and drug use - 1996 Supplementary survey report.

[38] Weinberg MK, Seton C, Cameron N (2018). The measurement of subjective wellbeing: item-order effects in the personal wellbeing index-adult. J Happiness Stud.

[39] Irvine K, Baker DF, Eyeson-Annan M (2004). Population health monitoring and surveillance: question development field testing -field test 3 report.

[40] Australian Bureau of Statistics (2018). Census of population and housing: socio-economic indexes for areas (SEIFA), Australia, 2016.

[41] VicHealth (2016). VicHealth Indicators Survey LGA Profiles: VicHealth.

[42] VicHealth (2016). Explore Your Data: VicHealth.

[43] Municipal Association of Victoria (2019). Vic Councils.

[44] Derrick B, Toher D, White P (2016). Why Welchs test is type I error robust. Quant Method Psychol.

[45] AAPOR on-line Response Rate Calculator 6. 2016. https://www.aapor.org/AAPOR_Main/media/MainSiteFiles/Response-Rate-Calculator-4-0-Clean-18-May-2016.xlsx Accessed 18 May 2016.

[46] Australian Bureau of Statistics (2013). Census 2011 Quick Stats: ABS.

[47] Yusuf F, Leeder SR (2015). Making sense of alcohol consumption data in Australia. Med J Aust.

[48] Capic T, Hutchinson D, Richardson B, Fuller-Tyszkiewicz M, Hartley-Clarke L, Cummins R (2015). Australian Unity Wellbeing Index: Report 32.0 The Wellbeing of Australians: Housing affordability.

[49] Australian Bureau of Statistics (2015). General Social Survey: User Guide, Australia, 2014.

[50] OECD (2015). Better Life Index - Edition 2015.

[51] Australian Institute of Health and Welfare (2016). National Drug Strategy Household Survey 2016: detailed findings.

[52] Powell BJ, Fernandez ME, Williams NJ, Aarons GA, Beidas RS, Lewis CC (2019). Enhancing the impact of implementation strategies in healthcare: a research agenda. Front Public Health.

