# Supplementary material for: The VicHealth Indicators population survey: methodology, prevalence of behavioural risk factors, and use in local policy
Source: BMC Public Health. 2020 Oct 2;20:1497. doi: 10.1186/s12889-020-09605-5 (PMC7531143; doi:10.1186/s12889-020-09605-5)
Supplement: Supplementary file 1 — Additional file 1. VicHealth Indicators survey questionnaire. Listing of all survey questions. [file 12889_2020_9605_MOESM1_ESM.docx]

**VicHealth Indicators Survey**

## INTRODUCTION & CONSENT

Good (morning/afternoon/evening). My name is (….) and I am calling on behalf of VicHealth from the Social Research Centre, part of the Australian National University. We are conducting a major study on the health and wellbeing of Victorians to help us understand the needs of different communities.

I just need to mention that the interview takes around 15 minutes. Participation is completely voluntary and you can withdraw at any time. All data is kept confidential within the limits of the law. Would it be okay if we made a start now?

## MODULE A: GENERAL and MENTAL WELLBEING

1. Okay, thinking about your own life and your personal circumstances, how satisfied are you with your life as a whole? Please use a scale from 0-10, where 0 is completely dissatisfied and 10 is completely satisfied.
2. Record number
3. (Don’t know)
4. (Refused)
5. Turning now to various areas of your life. How satisfied are you with…?
6. … your standard of living?
7. ... your health?
8. … what you are currently achieving in life?
9. … your personal relationships?
10. … how safe you feel?
11. … feeling part of your community?
12. … your future security?

*(RESPONSE FRAME)

1. Record number (Allowable range = 0 to 10)
2. (Don’t know)
3. (Refused)
4. CD-RISC 2: Able to adapt to change*
5. CD-RISC 2: Tend to bounce back after illness or hardship*

****Full question text proprietary to CD-RISC 2 questionnaire. Cannot be reproduced here.***

1. On a scale of 1 to 7, where 1 is strongly disagree and 7 is strongly agree, do you agree or disagree that…?
2. People around here are willing to help their neighbours
3. This is a close-knit neighborhood
4. People in this neighbourhood can be trusted

*(RESPONSE FRAME)

1. 1 - Strongly disagree
2. 2
3. 3
4. 4
5. 5
6. 6
7. 7 – Strongly agree
8. (Don’t know / not stated)
9. (Refused)
10. How safe or unsafe do you feel when you are in the following situations? How safe do you feel...?
    1. At home by yourself during the day?
    2. At home by yourself after dark?
    3. Walking in your local area alone during the day?
    4. Walking in your local area alone after dark?

*(RESPONSE FRAME)

1. Very safe
2. Safe
3. Neither safe nor unsafe
4. Unsafe
5. Very unsafe
6. (Never alone in this situation)
7. (Don’t know)
8. (Refused)

## MODULE B: PHYSICAL ACTIVITY

1. Thinking now about physical activity… In a usual week, on how many days do you do a total of 30 min or more of physical activity, which was enough to raise your breathing rate?

This may include sport, exercise and brisk walking or cycling for recreation or to get to and from places, but should not include housework, gardening or physical activity that may be part of your job.

1. None
2. Number of days given (1 – 7)
3. (Not applicable)
4. (Don’t know)
5. (Refused)
6. What are the three main types of physical activities that you USUALLY do?
7. Aerobics
8. Airsports
9. Aqua aerobics
10. Archery
11. Athletics, track and field
12. Australian rules football
13. Badminton
14. Baseball
15. Basketball (indoor and outdoor)
16. Beach volleyball
17. Billiards/ snooker/ pool
18. Bocce/ boules/ petanque
19. Boxing
20. Bush walking
21. Canoeing/kayaking
22. Carpet bowls
23. Cricket (indoor)
24. Cricket (outdoor)
25. Croquet
26. Cross country running
27. Cycling/ BMXing
28. Dancing/ ballet
29. Darts
30. Fencing
31. Fishing
32. Fitness/ gym
33. Football sports
34. Frisbee/ boomerang throwing
35. Golf
36. Gymnastics
37. Hockey (indoor and outdoor)
38. Horse riding/ equestrian activities/ polo
39. Ice/ snow sports
40. Jogging/ running
41. Lawn bowls
42. Martial arts
43. Minigolf
44. Motor sports
45. Netball (indoor and outdoor)
46. Pilates
47. Racewalking
48. Rock climbing/ abseiling/ caving
49. Rope skipping
50. Rowing
51. Royal tennis
52. Rugby league
53. Rugby union
54. Sailing
55. Scuba diving/ snorkelling
56. Shooting sports
57. Skateboarding/ inline hockey/ roller sports
58. Soccer (indoor)
59. Soccer (outdoor)
60. Softball/ tee ball
61. Squash/ racquetball
62. Surf lifesaving
63. Surf sports
64. Swimming/diving
65. Table tennis
66. Tennis (indoor and outdoor)
67. Tenpin bowling
68. Trail bike riding
69. Triathlons
70. Volleyball (indoor and outdoor)
71. Walking
72. Waterskiing/ powerboating
73. Water volleyball/ canoe polo/ rafting/ other water sports
74. Weight lifting/ body building
75. Windsurfing/ sailboarding
76. Yoga
77. Other (Specify)
78. (Don’t know)
79. (Refused)
80. Is the [(name of sport/physical activity)] organised by a club, association or other organisation?
81. Yes
82. No
83. (Don’t know)
84. (Refused)
85. What type of club, association or organisation organised the [<name of sport/physical activity>]?
86. Fitness, leisure or indoor sports centre
87. Sports club or association
88. Recreation club or association (e.g., bushwalking club)
89. Work
90. Educational Institution (e.g., Tafe, University)
91. Physical activity courses
92. Private business (e.g., private personal training, pilates or yoga studio)
93. Community fitness programs/events
94. Other (specify)
95. (Don’t know)
96. (Refused)
97. Was the [<name of sport/physical activity>] competitive or non-competitive? By competitive I mean results are formally recorded, there is a ladder/league table to determine the winner, and an identified body under which the competition is run.
98. Competitive
99. Non-competitive
100. (Don’t know)
101. (Refused)
102. Who do you usually do the [<name of sport/physical activity>] with?
103. By yourself
104. With friends/family
105. Other (specify)
106. (Don’t know)
107. (Refused)
108. The following question is about sitting at work, including meal and snack breaks and time spent sitting at a desk. How much time do you spend sitting at work on a usual work day?
109. Time per day given in HOURS (Specify_____)
110. Time per day given in MINUTES (Specify_____)
111. Did not sit at work
112. I do not work
113. (Don’t know)
114. (Refused)

## MODULE C: HEALTHY EATING

1. Now some questions about food. How many serves of vegetables do you USUALLY eat each day - a ‘serve’ is ½ cup of cooked vegetables or 1 cup of salad vegetables.
2. Record number of serves PER DAY
3. (Don’t know)
4. (Refused)
5. How many serves of fruit do you USUALLY eat each day - a ‘serve’ is 1 medium piece or 2 small pieces of fruit or 1 cup of diced pieces.
6. Record number of serves PER DAY
7. (Don’t know)
8. (Refused)
9. How many cups of water do you usually drink in a day? 1 cup=250ml or a household cup. 1 average 600mL bottle of water = 2.5 cups.
10. Number of cups per day given (Specify_____)
11. Number of litres per day given (Specify______)
12. None (don’t drink water on a daily basis)
13. Other (SPECIFY)
14. (Don’t know)
15. (Refused)
16. How often do you eat take away meals and snacks that are bought from fast food or takeaway food outlets? Examples could be pizza, hamburgers, hot chips.
17. Most days (6-7 times per week)
18. 3-5 times per week
19. 1-2 times per week
20. 2-3 times per month
21. Once per month
22. Less than once per month
23. Never
24. (Don’t know)
25. (Refused)

## MODULE D: ALCOHOL

1. Have you had an alcoholic drink of any kind in the last 12 months?
2. Yes
3. No
4. (Don’t know)
5. (Refused)
6. How often do you drink five or more standard drinks in a single session? A standard drink is equal to 1 pot of full strength beer, 1 small glass of wine or 1 pub-sized nip of spirits.
7. Every day
8. 5-6 days a week
9. 3-4 days a week
10. 1-2 days a week
11. 2-3 days a month
12. About 1 day a month
13. Less often
14. Never
15. (Don’t know)
16. (Refused)
17. How often do you drink eleven or more standard drinks in a single session?
18. Every day
19. 5-6 days a week
20. 3-4 days a week
21. 1-2 days a week
22. 2-3 days a month
23. About 1 day a month
24. Less often
25. Never
26. (Don’t know)
27. (Refused)
28. The following question is about attitudes toward drinking alcohol.
29. Do you agree or disagree that your FAMILY AND FRIENDS think that getting drunk every now and then is okay? By getting drunk I mean drinking to the point of ‘losing balance’.
30. Do you PERSONALLY agree or disagree that getting drunk every now and then is okay?

*(RESPONSE FRAME)

1. Strongly agree
2. Agree
3. (Neither agree nor disagree)
4. Disagree
5. Strongly disagree
6. (Don’t know)
7. (Refused)

## MODULE E: GENDER EQUITY

1. The statements I’m about to read out describe different attitudes that people have. Please tell me whether you strongly agree, somewhat agree, somewhat disagree or strongly disagree.
2. Men should take control in relationships and be the head of the household
3. Women prefer a man to be in charge of the relationship

*(RESPONSE FRAME)

1. Strongly agree
2. Somewhat agree
3. (Neither agree nor disagree)
4. Somewhat disagree
5. Strongly disagree
6. (Don’t Know / Can’t Say)
7. (Refused)

## MODULE F: SOCIO-DEMOGRAPHICS

1. Now I have some questions to help us analyse the results. Just to confirm, what is your gender?
2. Male
3. Female
4. Other
5. (Refused)
6. How old were you last birthday?
7. Age given
8. (Refused)
9. ncluding yourself, how many people aged 18 years and over live in this household?
10. Number given (Specify)
11. (Don’t know)
12. (Refused)
13. Which of these BEST describes your household?
14. Couple only
15. Couple with dependent child / children
16. Couple with non-dependent child / children
17. Couple with dependent and non-dependent children
18. One parent family with dependent child / children
19. One parent family with non-dependent child / children
20. One parent family with dependent and non-dependent children
21. Group household
22. One person household, or
23. Something else (Specify)
24. (Don’t know)
25. (Refused)
26. How many children, if any, live in your household. This includes children over 18 years?
27. None
28. Number of children given (specify)
29. (Don’t know)
30. (Refused)
31. What is the age of each child, starting with the youngest?
32. Age given (specify)
33. (Don’t know)
34. (Refused)
35. Are you a parent of anyone in this household?
36. Yes
37. No
38. (Don’t Know)
39. (Refused)
40. Are you of Aboriginal or Torres Strait Islander origin?
41. No, not Aboriginal or Torres Strait Islander
42. Yes, Aboriginal
43. Yes, Torres Strait Islander
44. Yes, Aboriginal and Torres Strait Islander
45. (Don’t know)
46. (Refused)
47. Which of the following options best describes how you think of yourself?
48. A) Straight (heterosexual)
49. B) Gay or lesbian
50. C) Bisexual, or
51. D) Other
52. (Don’t Know)
53. (Refused)
54. In which country were you born?
55. Australia (includes External Territories)
56. United Kingdom (incl. England, Scotland, Wales, Northern Ireland)
57. New Zealand
58. Italy
59. Greece
60. China
61. Vietnam
62. Lebanon
63. Other (Specify)
64. (Don’t know)
65. (Refused)
66. Do you speak a language other than English at home?
67. No, English only
68. Yes, Italian
69. Yes, Greek
70. Yes, Cantonese
71. Yes, Mandarin
72. Yes, Arabic
73. Yes, Vietnamese
74. Yes, German
75. Yes, Spanish
76. Yes, Tagalog (Filipino)
77. Yes, Other (Specify)
78. (Don’t know)
79. (Refused)
80. What is the highest year of schooling you have completed?
81. Year 12 or equivalent
82. Year 11 or equivalent
83. Year 10 or equivalent
84. Years 7- 9 or equivalent
85. Completed primary school but did not go to high school
86. Some primary school only
87. Did not go to school
88. (Don’t know)
89. (Refused)
90. What is the highest post-school educational qualification that you have obtained?
91. No post school edicational qualifiaction
92. Certificate I or Certificate II
93. Certificate III or Certificate IV
94. Associate Diploma
95. Undergraduate Diploma
96. Bachelor Degree
97. Master’s Degree, Postgraduate Degree or Postgraduate Diploma
98. Doctorate
99. Other (specify)
100. (Don’t know)
101. (Refused)
102. Do you have a disability, health condition or injury that has lasted, or is likely to last, 6 months or more which restricts your everyday activities?
103. Yes
104. No
105. (Don’t know)
106. (Refused)
107. Which of the following ranges best describes your <personal / household’s> approximate income, from all sources, before tax is taken out, over the last 12 months?
108. Less than $10,000
109. $10,000 – less than $20,000
110. $20,000 – less than $30,000
111. $30,000 – less than $40,000
112. $40,000 – less than $50,000
113. $50,000 – less than $60,000
114. $60,000 – less than $80,000
115. $80,000 – less than $100,000
116. $100,000 – less than $125,000
117. $125,000 – less than $150,000
118. $150,000 – to less than $200,000
119. $200,000 or more
120. (Don’t know)
121. (Refused)
122. Do you have access to the Internet at home, whether through a computer, mobile phone or other device?
123. Yes
124. No
125. (Don’t know)
126. (Refused)
127. Do you currently do any work in a job, business or farm?
128. Yes
129. No
130. (Don’t know)
131. (Refused)
132. How many hours in total per week do you usually work in your job / jobs…?
133. Hours given (specify)
134. (Don’t know)
135. (Refused)
136. Which of these best describes your current main activity? Are you…?
137. Self employed
138. Employed for wages, salary or payment in kind
139. Unemployed
140. Engaged in home duties
141. A student
142. Retired,
143. Unable to work, or
144. Something else (Specify)
145. (Don’t know)
146. (Refused)
147. What has been your MAIN occupation for most of your life?
148. Managers
149. Professional
150. Technician or trades worker
151. Community or personal service worker
152. Clerical or administrative worker
153. Sales worker
154. Machinery operator or driver
155. Labourer
156. Other (Specify)
157. Have not worked
158. (Don’t know)
159. (Refused)
160. Which of the following best describes your current marital status? Are you…
161. Married
162. Living with a partner
163. Widowed
164. Divorced
165. Separated, or
166. Never married
167. (Don’t know)
168. (Refused)
